# Positive effects of COVID-19 lockdown on river water quality: evidence from River Damodar, India

**DOI:** 10.1038/s41598-021-99689-9

**Published:** 2021-10-11

**Authors:** Baisakhi Chakraborty, Biswajit Bera, Partha Pratim Adhikary, Sumana Bhattacharjee, Sambhunath Roy, Soumik Saha, Anitabha Ghosh, Debashish Sengupta, Pravat Kumar Shit

**Affiliations:** 1 PG Department of Geography, Raja N. L. Khan Women’s College (Autonomous), Gope Palace, Midnapore, West Bengal 721102 India; 2 Department of Geography, Sidho Kanho Birsha University, Purulia, India; 3 ICAR Indian Institute Water Management, Bhubaneswar, Odisha 751023 India; 4 Department of Geography, Jogesh Chandra Chaudhuri College, University of Calcutta, 30, Prince Anwar Shah Rd, Badam Talla, Tollygunge, Calcutta, West Bengal 700033 India; 5 Department of Geography, University of Calcutta, 35, Ballygunge Circular Road, Ballygunge, Calcutta, 700019 India; 6 Department of Geology and Geophysics, Indian Institute of Technology (IIT), Kharagpur, West Bengal 721302 India

**Keywords:** Ecology, Environmental sciences

## Abstract

The global economic activities were completely stopped during COVID-19 lockdown and continuous lockdown partially brought some positive effects for the health of the total environment. The multiple industries, cities, towns and rural people are completely depending on large tropical river Damodar (India) but in the last few decades the quality of the river water is being significantly deteriorated. The present study attempts to investigate the river water quality (RWQ) particularly for pre- lockdown, lockdown and unlock period. We considered 20 variables per sample of RWQ data and it was analyzed using novel Modified Water Quality Index (MWQI), Trophic State Index (TSI), Heavy Metal Index (HMI) and Potential Ecological Risk Index (RI). Principal component analysis (PCA) and Pearson’s correlation (r) analysis are applied to determine the influencing variables and relationship among the river pollutants. The results show that during lockdown 54.54% samples were brought significantly positive changes applying MWQI. During lockdown, HMI ranged from 33.96 to 117.33 with 27.27% good water quality which shows the low ecological risk of aquatic ecosystem due to low mixing of toxic metals in the river water. Lockdown effects brought river water to oligotrophic/meso-eutrophic condition from eutrophic/hyper-eutrophic stage. Rejuvenation of river health during lockdown offers ample scope to policymakers, administrators and environmentalists for restoration of river health from huge anthropogenic stress.

## Introduction

Presently, the world is facing an unprecedented disaster of the Covid-19 pandemic by the newly emerged coronavirus. Initially, it was reported in China and through the process of mutation; virus has gradually changed its behaviour as well as functionality. Since the outbreak of COVID-19 during the last quarter of 2019, more than 173 million people were affected globally and around 3.5 million deaths were reported by WHO^1^ up to last week of May, 2021. This stormy upheaval of COVID-19 pandemic brings emergency for public health, globally. The GDP of most of the countries have been fallen due to long term shutdown of different sectors of economy. Subsequently, global socio-economic and educational pillars have also been partially collapsed for such disastrous event. Most of the urban life has been confined into curbed cubicles due to strict lockdown.

India is the second highest populated country in the world and people are suffering from the extreme uncontrolled ruin of the Covid-19 pandemic since January, 2020. Rapid transmission of this viral infection affected more than 28 million people and took near about 330,000 lives upto last week of May, 2021 (WHO). This number of mortal cases has crossed over a huge unpredictable position in its initial stage, if there was no restriction of social distancing among people. On this circumstance Government of India took an unprepared decision of sudden full ‘lockdown’ to its whole country effected from 25th March, 2020 to 31st May 2020^2^. The economic progress of Government and private sectors was very badly hampered due to countrywide full lockdown but the spread of infection among the people has been reduced. Environmental researchers discovered that almost four months of constant lockdown brought very positive changes of environmental quality through closing of industries, transport and business activities. However, this result was reflected not only in India, but also all other countries due to maintenance of lockdown process properly^3–12^.

In the recent years, degradation of aquatic environment, river water quality (RWQ), pollution and health, river ecosystem services etc. has been amplified due to rapid urbanization, industrialization and execution of various developmental activities. The majority of the large world rivers are polluted by anthropogenic activities such as non degradable agriculture fertilizers and untreated industrial sewage discharge into rivers^13^. The river water quality is also declined gradually through toxic materials and excess nutrients supply from agrarian fields^14^. CPCB^15^ reported that around 38,000 million liters per day untreated sewage and wastewater are discharged into the Indian River systems. Due to limited sewage treatment capacity, around 52% of untreated sewage and industrial effluents (integrated spared pollution) are discharged into Indian rivers^16^. RWQ parameters are typically spatiotemporal variables and it is very difficult to measure because constant change of attributes and severe anthropogenic stress. In the Covid-19 lockdown situation, toxic materials and untreated industrial sewage discharge into rivers are bunged due to limited anthropogenic activities. That time, water quality assessment and monitoring are more important for the identification of pollution sources, pollution level and types of anthropogenic stress.

Many scholars have studied on impact of COVID-19 lockdown on river water quality using WQI method^3–6,10,11^. Karunanidhi et al.^4^ showed the effects of COVID-19 lockdown on microbial and metals contaminations in a part of Thirumanimuthar River, South India. Khan et al.^5^ reported that possible risk of faecal-oral transmission of corona virus was creating a major concern of the residents across Gomti river stretch in Lucknow city (India). However, lockdown of Covid-19 helped to ameliorate the river water quality (RWQ) across the world reported by many researchers. Yunus et al.^2^ showed a significant decrease of suspended particulate matter (SPM) in Vembanad Lake, Kerala (India) during lockdown period. Arif et al.^17^ reported a significant reduction in Yamuna River water pollution during the lockdown phase compared with the pre-lockdown phase. An assessment on river Ganga revealed that no admixing of urban and industrial effluents has brought positive improvement on river water quality in term of particular parameters at many sites of its course^18–25^. A recent study was conducted in an industrial catchment of river Damodar, India and it assesses the impact of lockdown on the change of water quality^10,11^. Qiao et al.^26^, evaluated the trend of surface water quality from 2000 to 2019 and they assessed the effects of Covid-19 lockdown on Yangtze River Basin (China). Dobson et al.^27^, developed an integrated model to evaluate the pollution level of tributaries of the River Thames (London) under the Covid-19 situation.

Damodar is a very important tropical river and it also sustains the society along with vast economic activities and ecological diversity within its catchment area. However, there is no previous comprehensive study of Covid-19 lockdown effects on river water quality, nutrient status, toxic heavy metals and their ecological risk impact on aquatic environment of a tropical river Damodar, India.

Therefore, the present study attempts (1) to analyse water quality, nutrient tropic level, toxic heavy metals, and ecological risk using different indexing methods in three phases (pre-lockdown, lockdown, and unlock); (2) to evaluate the differences in concentration of twenty RWQ variables for the lockdown compared with pre-lockdown and unlock phases, 2020, thereby to assess the impact of Covid-19 lockdown on Damodar river water quality, nutrient level, aquatic toxicity, and ecological status and finally, (3) to outline a framework of possible remedial management strategies.

## Materials and methods

### Study area

**Figure 1:**
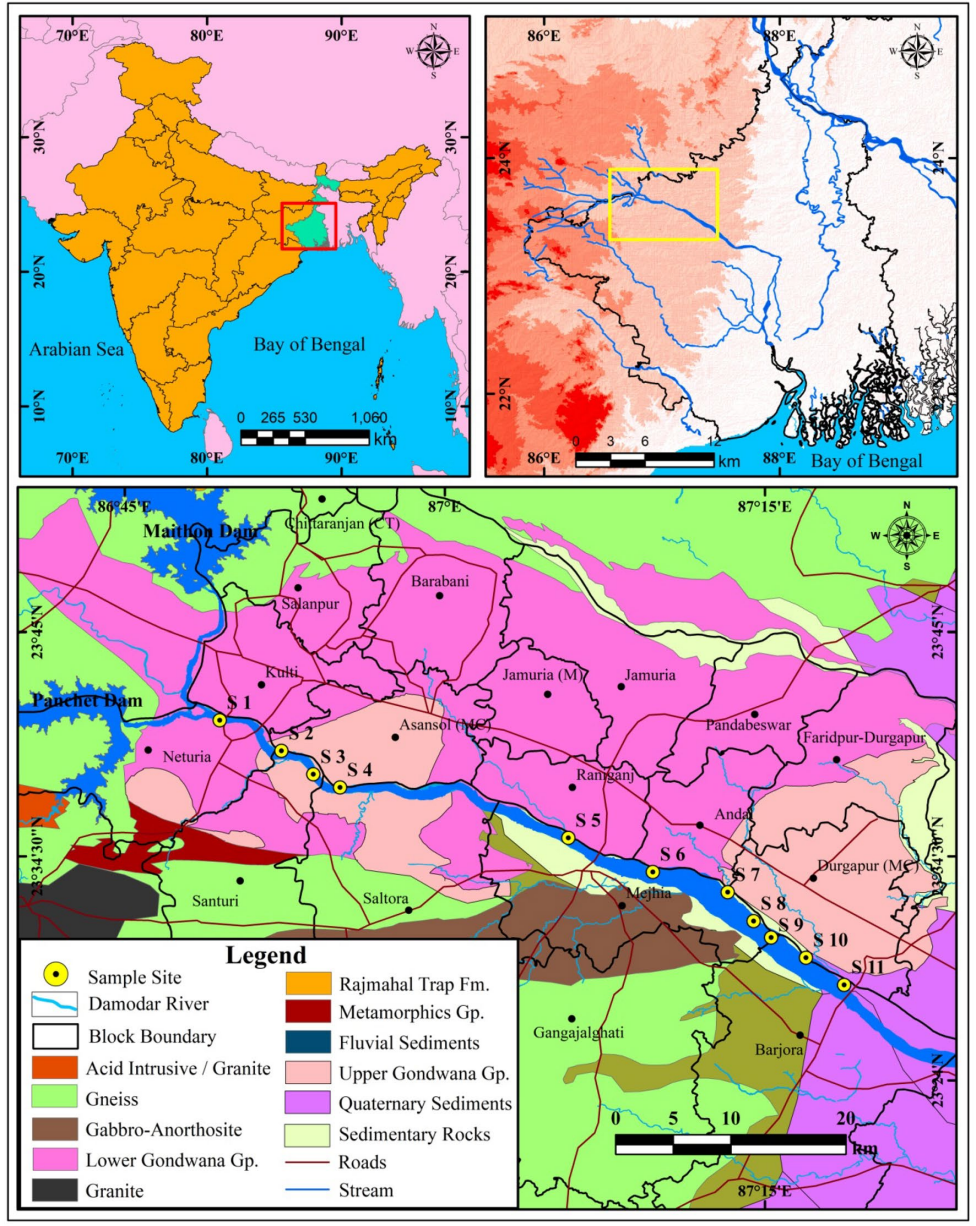
Location map of the study stretch of a tropical river Damodar (India). The diagram is prepared by open source QGIS 3.16 software (https://qgis.org/en/site/forusers/download.html).

The important river Damodar (563 km) originates from Khamarpat hill under Palamau district of Jharkhand state (India). It flows toward east direction and ultimately it joins with river Bhagirathi-Hooghly in West Bengal. Upper and middle parts of the river basin have rich diversity of minerals and standard quality coal reserve of Gondwana formations. Abundant supply of fresh river water with high mineral and energy resources attracts many large, medium and small-scale industries since historical time. River Damodar is the principal supplier of water resource to drinking, industrial and domestic purpose in its catchment area. Therefore, such favourable environment attracts huge population along with industrial integration in this area. The present study area is bounded by 23° 28′ 28.7″ N to 23° 40′ 52.5″ N and 86° 49′ 26.8″ E to 87° 18′ 42.4″ E and 65.37 km river stretch has been selected for the study. In this section high, agglomeration of industries and allied human works intensively developed along the riverside. Many iron and steel plants, thermal power plant, sponge iron factory, chemical industries, coal mining fields and urban centres have been developed through the evolution of time. As a result, huge untreated waste (solid/ liquid), hot water, coal dust and urban effluents are being regularly discharged to the riverbed through various connecting channels which are locally called *nallas* (Fig. 1)^10,11^.

### Sample collection and data analysis

Water samples were collected from eleven discharged points of industrial effluents on main riverbed. First, samples were taken on December 2019 (pre-lockdown/ normal period), again second, samples were collected in June, 2020 (during lockdown) to assess the changes on river water quality due to temporarily closing of industries. Third, samples were obtained in November, 2020 (after unlock phase) to get clear idea about effects of industries on the river water quality. Samples were obtained from 0.5 m below the surface water level within 5 m influencing radius zone. Pre cleaned polyethylene bottles (500 ml) were used for the collection of five subsamples from each sampling site and mixed up to get a bulk contain (1 l). All samples were carried properly for further analysis in laboratory. Sample containers were labelled as S1, S2, S3… to S11 for properly identification (Fig. 1). Total 20 parameters were analysed from each sample of each period. Important parameters such as pH, electrical conductivity (EC), total dissolved solids (TDS), turbidity, magnesium (Mg^2+^), calcium (Ca^2+^), chloride (Cl^-^), sulphate (SO_4_^2–^), nitrates (NO_3_^−^), Biological Oxygen Demand (BOD), Dissolved Oxygen (DO), zinc (Zn^2+^), cadmium (Cd^2+^), lead (Pb^2+^), nickel (Ni^2+^), chromium (Cr), iron (Fe^2+^), chlorophyll *a* (Chl*a*), total phosphorus (TP), and Secchi disk depth (Sd) have been considered. Consequently, pH and EC were measured at the sampling sites using Thermo probe, Hanna HI9811-5 potable meters respectively. DO was determined through Winkler’s method at the sampling spot immediately^28^. EC denoted by microsiemens per centimetre. TDS was determined following the procedure given by Hem (1991). Turbidity was denoted by Nephelometric Turbidity Unit (NTU’s). All cation, anions, BOD and DO were expressed in mg/l while all heavy metals, TP and Chl*a* denoted as microgram/l. All other physico-chemical parameters and heavy metals were analysed by standard procedure which was prescribed by American Public Health Association (APHA)^29^. Chl*a* and total phosphorus were estimated following APHA^29^ standard procedures. Secchi disk (Sd) with 8 in. diameter and attached cord in disk centre was used for depth measurement and expressed in meters at the maximum limit of depth where disk was seen from the above into the water.

### Modified water quality index (MWQI)

MWQI of the 33-sample water was conducted for 11 sample sites by important water quality parameters namely pH, TDS, EC, turbidity, Mg^2+^, Ca^2+^, Cl^-^, SO_4_^2–^, NO_3_^–^, BOD and DO. We considered 11 variables per sample in the index. The calculation of MWQI was conducted following the method of Vasistha and Ganguly^30^.

At first, pre defined weightage was assigned for each selected parameter. The weightage of each parameter was obtained from previous literatures. After that, relative weight of each parameter was derived by the formula.

1 $$ RW = AW/\sum\limits_{i = 1}^{n} {AW} $$

where RW is relative weight of each parameter, AW is assigned weight obtained from past literature (AW of pH = 1, TDS = 1.79, EC = 1.78, turbidity = 1.09, Ca^2+^  = 0.8, Mg^2+^  = 0.72, Cl^–^ = 1.28, SO_4_^2–^ = 1.60, NO_3_^–^ = 2.32, BOD = 1.72, DO = 2.85) and n is total number of parameters considered for analysis.

Second, quality assessment (Q_i_) of each parameter was obtained following the formula.

2 $$ Q_{i} = (C_{i} \times S_{i} ) \times 100 $$

where C_i_ is concentration of particular parameter in sample water, S_i_ is standard permissible limit of each parameter as suggested by BIS^31^ and WHO^31^ (Table 1).

**Table 1:** Descriptive statistics of twenty variables of physio chemical, heavy metals and biological parameters in three period.

Parameters	Pre-lockdown	Lockdown phase			Unlock phase			Permissible limit	
	Mean ± SD	Mean ± SD	Mean diff (%)	SD diff (%)	Mean ± SD	Mean diff (%)	SD diff (%)	Standard value	Guideline by
pH	7.46 ± 0.38	6.92 ± 0.48	− 7.24	26.32	7.42 ± 0.49	− 0.54	28.95	6.5	BIS (2012)
TDS (mg/l)	740.65 ± 50.24	524.85 ± 26.29	− 29.14	− 47.67	589.96 ± 48.30	− 20.35	− 3.86	500	WHO (2011)
Turbidity (NTU/l)	20.73 ± 4.42	5.63 ± 3.52	− 72.84	− 20.36	11.36 ± 5.66	− 45.20	28.05	1	BIS (2012)
EC (µg/l)	1157.27 ± 78.50	820.09 ± 41.09	− 29.14	− 47.66	921.82 ± 75.47	− 20.35	− 3.86	300	BIS (2012)
Mg^2+^ (mg/l)	70.36 ± 11.00	27.00 ± 3.00	− 61.63	− 72.73	46.81 ± 4.97	− 33.47	− 54.82	30	BIS (2012)
Ca^2+^ (mg/l)	131.09 ± 34.13	64.81 ± 9.48	− 50.56	− 72.22	99.54 ± 23.89	− 24.07	− 30.00	75	WHO (2011)
Cl^−^ (mg/l)	407.27 ± 65.13	198.18 ± 17.21	− 51.34	− 73.58	264.54 ± 27.69	− 35.05	− 57.49	250	WHO (2011)
So_4_^−^ (mg/l)	358.18 ± 41.19	127.27 ± 34.37	− 64.47	− 16.56	199.09 ± 68.62	− 44.42	66.59	200	BIS (2012)
NO_3_^−^ (mg/l)	86.45 ± 9.60	37.46 ± 5.48	− 56.67	− 42.92	64.23 ± 15.77	− 25.70	64.27	45	WHO (2011)
BOD (mg/l)	12.54 ± 3.47	7.27 ± 2.41	− 42.03	− 30.55	9.81 ± 2.71	− 21.77	− 21.90	5	BIS (2012)
DO (mg/l)	4.87 ± 0.49	7.29 ± 0.60	+ 49.69	22.45	6.344 ± 5.01	+ 32.24	12.24	6	BIS (2012)
Zn^2+^ (mg/l)	39,845.45 ± 6280.34	5300.00 ± 762.88	− 86.70	− 87.85	7566.36 ± 739.20	− 81.01	− 88.23	15,000	BIS (2012)
Cd^2+^ (mg/l)	10.20 ± 1.77	3.10 ± 1.33	− 69.61	− 24.86	5.01 ± 1.28	− 50.88	− 27.68	10	BIS (2012)
Pb^2+^ (mg/l)	26.18 ± 5.23	3.53 ± 1.66	− 86.52	− 68.26	5.36 ± 1.56	− 79.53	− 70.17	50	BIS (2012)
Ni^2+^ (mg/l)	84.55 ± 33.57	6.54 ± 1.63	− 92.26	− 95.14	22.36 ± 4.20	− 73.55	− 87.49	20	BIS (2012)
Cr (mg/l)	87.67 ± 10.32	33.85 ± 6.33	− 61.39	− 38.66	41.15 ± 7.98	− 53.06	− 22.67	50	WHO (2011)
Fe (mg/l)	674.82 ± 112.96	132.90 ± 8.67	− 80.31	− 92.32	284.36 ± 83.46	− 57.86	− 26.12	300	WHO (2011)
Sd (mts)	2.24 ± 0.45	2.79 ± 0.46	+ 24.55	2.22	2.55 ± 0.43	+ 13.84	− 4.44	–	–
Chl-a (mg/l)	76.09 ± 16.85	29.18 ± 9.00	− 61.65	− 46.59	49.36 ± 8.86	− 35.13	− 47.42	–	–
TP (mg/l)	62.45 ± 17.32	18.36 ± 6.10	− 70.60	− 64.78	33.00 ± 7.12	− 47.16	− 58.89	–	–

*SD* standard deviation, *BIS* Bureau of Indian Standards, *WHO* World Health Organization.

Qi for pH and DO was obtained through some modification of Eq. (1.2) because optimum concentration of these two parameters are little different from others. The optimum value of pH and DO is considered as 7.0 and 14.6 mg/l (100% saturation at 23 °C), respectively^32^. Thus, Q_i_ for these two parameters were performed using the formula.

3 $$ Q_{i} = (\frac{{C_{i} - V_{i} }}{{S_{i} - V_{i} }}) \times 100 $$

where V_i_ denotes optimum values of pH and DO.

Third, in this step sub index (SI_i_) was calculated for each considered parameter by multiplication of relative weight (RW) with quality assessment (Q_i_) value of each parameter using formula below.

4 $$ SI_{i} = RW \times Q_{i} $$

At last, MWQI was obtained for each sample site by summation of SI_i_ of each parameter as below:

5 $$ MWQI = \sum\limits_{i = 1}^{n} {SI_{i} } $$

Water quality (based on MWQI values) has been categorised into 5 classes such as excellent (≤ 50), good (50–100), poor (100–200), very poor (200–300) and unfit for drinking (≥ 300) as suggested by BIS^31^ (IS:10500).

### Heavy metal index (HMI)

Analysis of heavy metal index was done using 6 parameters as Cd^2+^, Zn^2+^, Cr, Pb^2+^, Ni^2+^, and Fe^2+^. Calculation was conducted through this formula^33^.

6 $$ Wi = K/Si $$

where W*i* suggests weightage of *i*th parameter, K means constant value (1), S*i* means standard value of *i*th parameter as per BIS^31^, and WHO^32^. In the next step, sub index calculation (Q*i*) was done through this formula.

7 $$ Qi = \sum\limits_{i = 1}^{n} {\frac{Mi}{{Si}}} \times 100 $$

where M*i* is the value of heavy metal concentration in sample water, S*i* is maximum limit of permissible of *it*h parameter in µg/l according to BIS^31^ and WHO^32^ (Table 1). At last, HPI was calculated using this formula which is given below.

8 $$ HPI = \frac{{\sum\limits_{i = 1}^{n} {WiQi} }}{{\sum\limits_{i = 1}^{n} {Wi} }} $$

where n indicates total number of parameters used for calculation of HPI. HPI can be classified into five categories such as excellent (0–25), good (26–50), poor (51–75), very poor (75–100) and unfit for drinking (> 100).

### Potential ecological risk (RI)

To assess the environmental response of heavy metal contamination, a new index was applied from sedimentological perspective and it was proposed by Hakanson^33^. In this method, effects of heavy metals on environment and possibilities to ecological risk can be determined by a single contamination coefficient, toxic response coefficient of heavy metals and comprehensive contamination of metals for any aquatic or soil environment using this formula^34^.

9 $$ C_{f}^{i} = C_{s}^{i} /C_{n}^{i} ,\;c = \sum\limits_{i = 1}^{n} {C_{f}^{i} } $$

10 $$ E_{r}^{i} = T_{r}^{i} \times C_{f}^{i} ,\;RI = \sum\limits_{i = 1}^{m} {E_{r}^{i} } $$

where C_s_^i^ specifies heavy metal contamination value, C_n_^i^ indicates reference value of heavy metals, C stands for degree of contamination by toxic heavy metals, E_r_^i^ represents ecological risk factor of any single substance, T_r_^i^ indicates ‘Toxic- response’ of any particular metal and RI denotes potential ecological risk index of all measured toxic metals. In this study, reference value of heavy metals was taken from standard preindustrial values of heavy metals as Cd = 1.0, Pb = 70, Cr = 90 and Zn = 175. Toxic response of heavy metals was used as follows: Cd = 30, Pb = 5, Cr = 2 and Zn = 1 (Hakanson^33^). Values of RI can be classified into four categories such as Practically uncontaminated (< 150), Moderately contaminated (150–300), Heavily contaminated (300–600), and Extremely contaminated (> 600).

### Trophic State Index (TSI)

Trophic status of river was identified by Trophic State Index (TSI) considering three parameters such as Secchi disk depth (Sd), Chlorophyll-*a* (Chl*a*), Total phosphorus (TP). Trophic State Index (TSI) was calculated by Carlson method^35^.

11 $$ TS(Sd) = 60.0 - 14.41 \times Ln(Sd) $$

12 $$ TS(TP) = 14.42 \times Ln(TP) + 4.15 $$

13 $$ TS(Chla) = 30.6 + 9.81 \times Ln(Chla) $$

14 $$ {\text{TSI }}\left( {\text{Trophic State Index}} \right) = \left[ {TS(Sd) + TS(TP) + TS(Chla)} \right]/3 $$

Values of TSI were classified into seven categories such as low oligotrophic (< 30), high oligotrophic (30–40), mesotrophic (40–50), (Mesotrophic), low eutrophic (50–60), medium eutrophic (60–70), high eutrophic (70–80), and very high eutrophic (> 80).

### Statistical and spatial analysis

A meta analysis such as descriptive statistics, Pearson correlation coefficient, analysis of variance (ANOVA test), principal component analysis (PCA) of all physico-chemical parameters, biological and heavy metals were applied to quantify the significant changes in three phases using least significant difference (LSD) at 0.05 level. All statistical analysis has been performed using SPSS 20 and MS-excel software while R programming language v. R 4.1.1 is used only for diagrammatic presentation. Inverse Distance Weightage (IDW) technique was performed on QGIS v.3.16 software for revealing spatial variation of water quality in three periods on the basis of different indexing method.

## Results

### Hydro-chemical properties of river water

**Figure 2:**
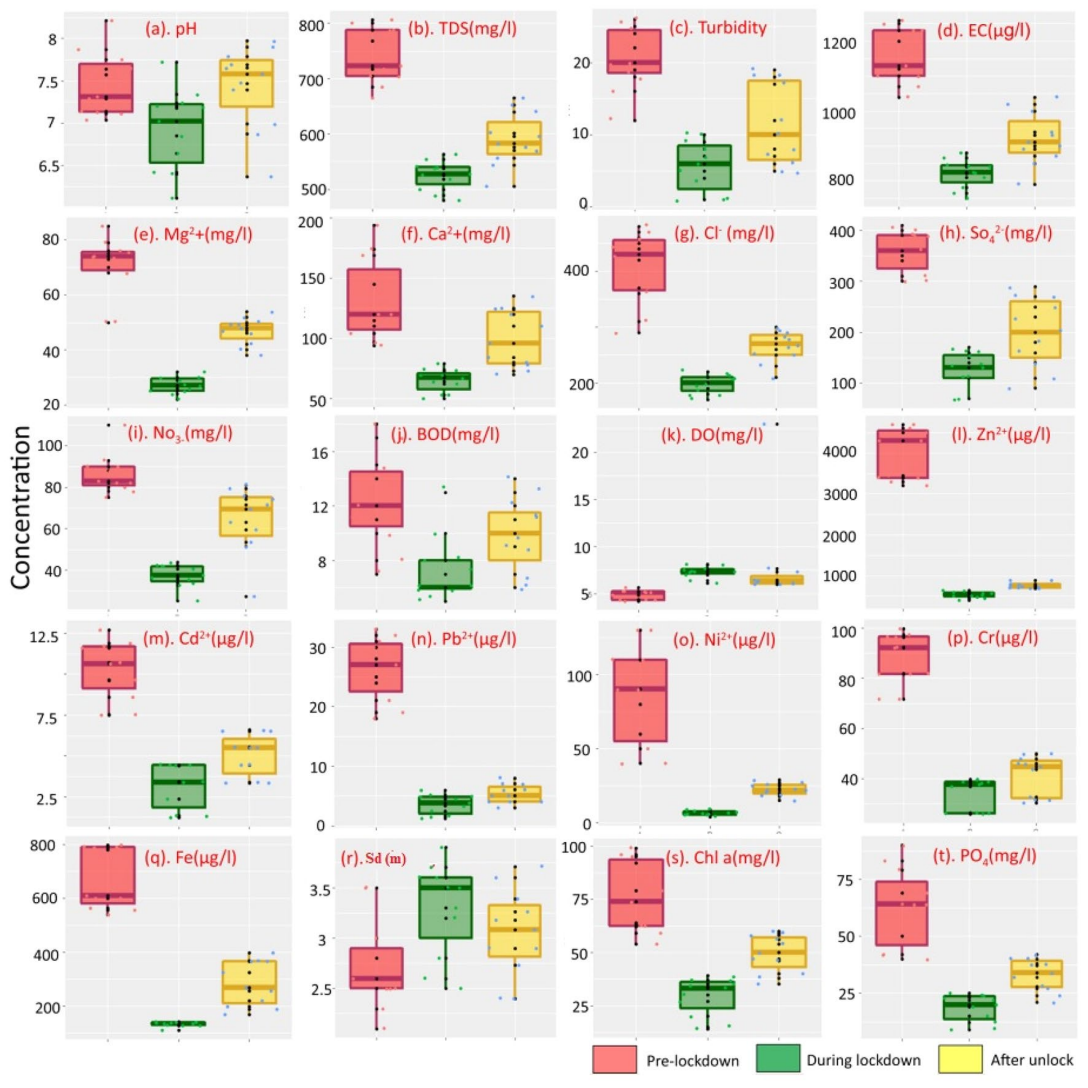
Box and whisker plot of different cations, anions and heavy metals concentration in three periods. This diagram is prepared by open. source R 4.1.1 software (https://cran.r-project.org/bin/windows/base/).

Table 1 represents the phase-wise mean, standard deviation, and percentage of mean distribution of hydro-chemical properties of RWQ. Box and whisker plots showed comparative changes pattern of each cation, anion, and heavy metals of three phases in Fig. 2. The maximum variables of RWQ were declined during the lockdown phase except DO and Sd (Fig. 2). In lockdown and unlock phases, Sd was increased by 24.55% and 13.84% respectively. DO was increased by 49.69% during the lockdown phase and around 32.24% during unlock phase respectively. The lowest Turbidity was observed during lockdown phase with a mean and SD of 5.6 ± 3.5 and it is declined by − 72.84% and − 45.84% during lockdown and unlock phase respectively. In cation, Mg^2+^ was dropped maximum of − 61.63% during lockdown phase and again − 33.47% was declined compared with pre-lockdown phase (December 2019). The SO_4_^2–^ decreases by − 64.47% and − 44.42% during lockdown and unlock phase respectively. For heavy metal like Ni^2+^-around 92.26% was declined during lockdown phase and around − 73.55% during unlock phase respectively. Chl-a (mg/l) was declined by − 61.65% during lockdown phase and rose to − 35.13% during unlock phase compared to pre-lockdown phase (December 2019). However, pre-lockdown phase, all physical, chemical and biological parameters were noticed higher than their permissible limit as suggested by WHO, 2011 and BIS, 2012 (Table 1), but most of the variables were dropped during lockdown phase and after that gradually increased during unlock phase.

Pearson’s correlation of metadata of RWQ parameters of three phases are shown in Table 2. Pre-lockdown phase showed that there are significant correlations among pH with all other parameters except Sd (Table 2a). Sd represented a very high significant negative correlation with pH, TDS, EC, Ca^2+^ and DO in water samples. A high negative correlation has been found for Sd with turbidity, Mg^2+^, BOD, Zn^2+^, Ni^2+^ and Fe^2+^. Significant negative correlation showed for Sd with NO_3_^−^ in pre-lockdown water samples. Cl^−^, SO_4_^–2^, Cd^2+^ and Cr indicated negative correlation but no such significance at any level with Sd. This correlation of Sd with all other parameters clearly suggested that the increasing of these components in river water quality directly helped to decrease visibility or clearance of water bodies. TDS was strong positively correlated with EC at 0.001 level of significance because electric conductivity directly dependent on the presence of TDS in water. Heavy metals indicated very high positive correlation with each other and supply of these components showed the common source of origin (industry, domestic wastes, crop field etc.). During the lockdown phase, there was no such significant correlation of heavy metals, Chl-*a*, and TP with pH, TDS, EC, Ca^2+^ in river water which brought low positive correlation among them (Table 2b). DO and Sd represent negative correlation with all parameters except each other and it clearly showed that the decreasing trend of all other parameters increases visibility of water body. In this phase, six heavy metals showed positive correlation with each other at the significant levels at 0.005. Turbidity showed very high correlation with NO_3_^−^, heavy metals, chlorophyll *a* and TP in river water. In this session, there were low positive correlations among pH, TDS, EC, Ca^2+^ with heavy metals in sample water with no significance. Correlation analysis of unlock phase showed that there were very high positive correlation of turbidity with anions, cations, BOD, heavy metals, chlorophyll, TP in water samples. The increasing trend of these components highly amplifies the turbidity of river water (Table 2c). Similarly, DO showed a very low positive correlation with all other parameters while Sd showed a very high negative correlation with turbidity and ions in river water quality.

**Table 2:**
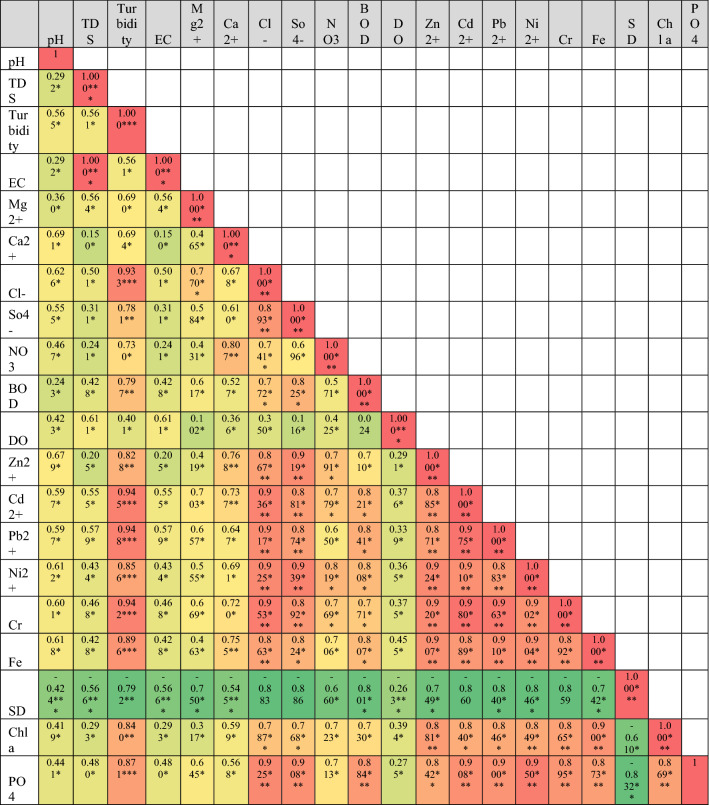

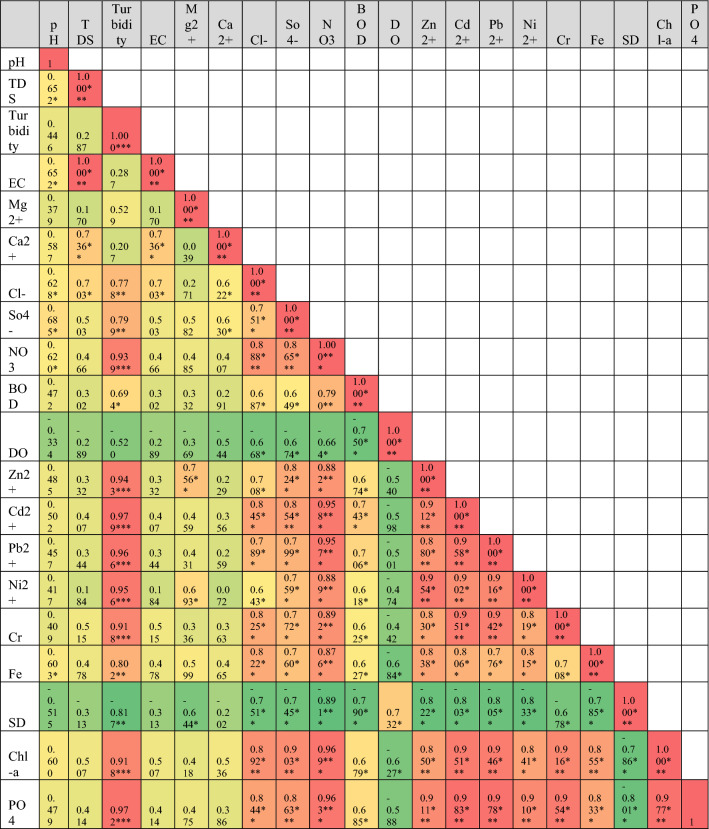

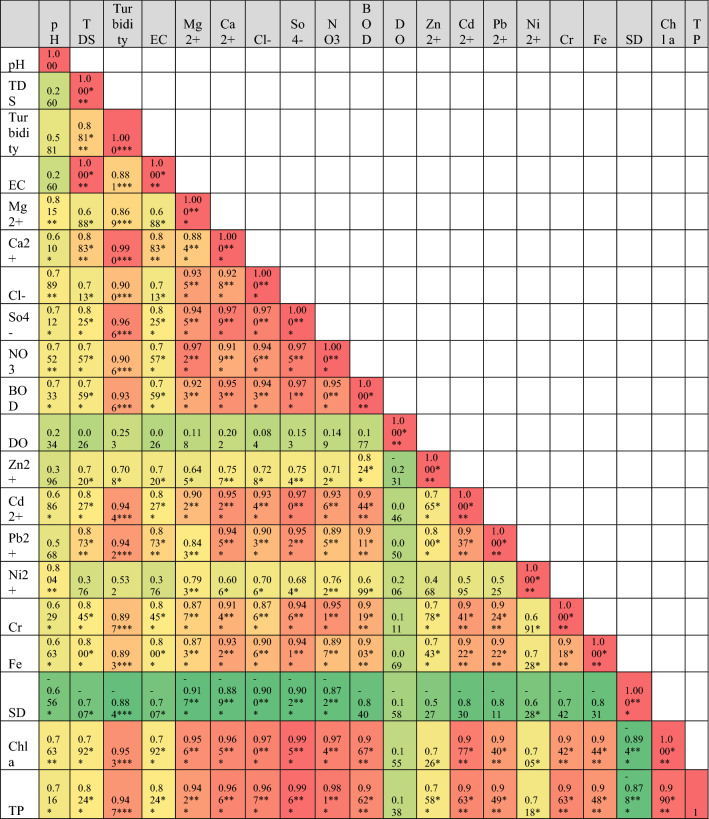
Pearson correlation matrix of water quality parameters (significant at p < 0.05*, p < 0.01**, p < 0.001***), (a) pre-lockdown (November 2020); (b) during-lockdown (July 2020) and after unlock phase (November 2020).

Red colour =  + positive relation; green colour = -negative relation.

The analysis of variance (ANOVA) was carried out for twenty RWQ parameters of pre-lockdown, during lockdown and unlock phase to test significant difference between variables and within variables. Average of 20 variables of three phases were compared by the least square difference (LSD) method at ρ < 0.05 level of significance. If the ‘F’ value of any variable scored above acceptance level (ρ < 0.05) then the result indicates significant changes of variables between groups by their means and rejection of the null hypothesis. Table S1 showed that all parameters have a significant change in three phases by their F values. F values of pH, BOD, DO and Sd were significant at 0.015%, 0.001%, 0.047% and 0.025% level respectively, which are much below than ρ = 0.05. All other variables showed their F value at 0.0001 level of significance i.e., there was absolute significant difference of variables among pre-lockdown, during lockdown and unlock phases by their mean (Table S1).

Principal component analysis (PCA) has been performed considering 20 RWQ parameters for identification of influencing component and quality variation among pre-lockdown, during lockdown and unlocks phases. Varimax rotated component matrix of pre-lockdown phase showed Eigen value of 3 factors having greater than one (Table S2). Factor 1 with 70.07% total variance indicated very high positive loadings of NO_3_^−^, PO_4_, Cl^−^, Cd^2+^, Ca^2+^, Zn^2+^, Sd, Ni^2+^, Pb^2+^, turbidity, DO, Cr, Chl-*a* in sample water. It showed very high supply of untreated waste water and solid materials to the riverbed. During lockdown, Factor 1 with 68.617% total variance showed very high positive loading of Pb^2^, PO_4_, turbidity, Cr, Cd, Ni^2+^, Chl *a*, No_3_^−^, Zn^2+^, Cl^−^, SO_4_^2–^, Fe in water samples which brought high activity of heavy metals. In unlock phase, PCA was performed and three components were extracted having Eigen value greater than 1. Factor 1 of 79.331% total variance showed that all parameters have very high positive loading except negative loading of Sd and EC. DO has very low positive loading in this phase. Factor 2 with 8.192% total variance showed positive loading of all parameters and positive correlation with each other except EC and Sd. Factor 3 with 5.099% total variance showed negative loading of Zn^2+^. DO showed very high positive loading to other parameters (Table S2).

### Water quality assessment by Modified Water Quality Index (MWQI)

**Figure 3:**
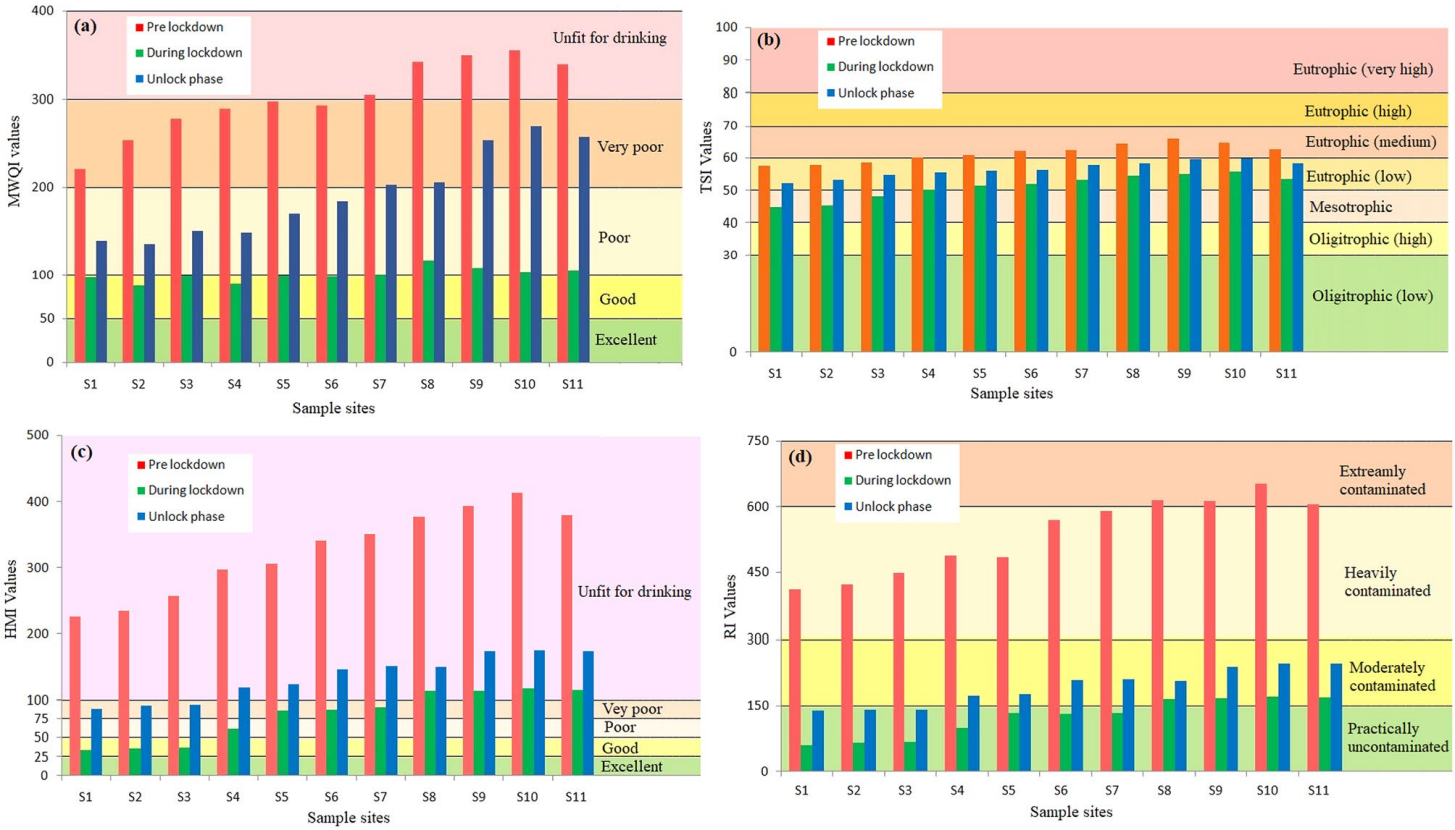
Spatiotemporal variation of water quality values at each sampling sites. This figure is prepared by MS office 2007 (https://microsoft-office-2007.en.softonic.com/).

Assessment of water quality by MWQI method showed significant changes in river water quality in three phases. MWQI of pre-lockdown phase was ranged from 254.19 to 354.96 with 54.54% very poor and 45.45% unfit for drinking water (Tables 3, 4). During lockdown, MWQI values ranged from 87.94 to 116.84 with very significant positive changes in water quality which showed that around 54.54% water samples were good quality and about 45.45% samples were poor quality. In unlock phase, MWQI values ranged from 136.12 to 269.53 with 54.54% water samples were poor quality and around 45.45% were very poor (Fig. 3a). MWQI values decreased by − 67.35% and − 36.18% during lockdown and unlock phase respectively compared with pre-lockdown phase (December 2019) (Table 3). It indicates the deterioration of water quality in unlock period mainly due to mixing of industrial effluents.

**Table 3:** Values of MWQI, TSI, HMI and RI in three sessions.

Indices	Pre lockdown	Lockdown			Unlock phase		
	Mean ± SD	Mean ± SD	Mean diff (%)	SD diff (%)	Mean ± SD	Mean diff (%)	SD diff (%)
MWQI	310.37 ± 34.07	101.33 ± 8.45	− 67.35	− 303.20	198.07 ± 48.65	− 36.18	42.79
TSI	61.99 ± 2.63	51.89 ± 3.26	− 16.29	19.33	56.97 ± 2.14	− 8.10	− 18.63
HMI	335.29 ± 59.77	86.06 ± 31.28	− 74.33	− 91.08	140.01 ± 31.01	− 58.24	− 48.12
RI	537.45 ± 87.19	124.53 ± 44.20	− 76.83	− 97.26	194.91 ± 42.17	− 63.73	− 51.63

**Table 4:** Water samples under each category of MWQI, HMI, RI and TSI in three periods.

Water quality	Category	Pre lockdown (%)	During lockdown (%)	After unlock phase (%)
MWQI	Excellent	0	0	0
	Good	0	54.54	0
	Poor	0	45.45	54.54
	Very poor	54.54	0	45.45
	Unfit for drinking	45.45	0	0
TSI	Oligotrophic (low)	0	0	0
	Oligotrophic (high)	0	0	0
	Mesotrophic	0	27.27	0
	Eutrophic (low)	36.36	72.72	100
	Eutrophic (medium)	63.63	0	0
	Eutrophic (high)	0	0	0
	Eutrophic (very high)	0	0	0
HMI	Excellent	0	0	0
	Good	0	27.27	0
	Poor	0	9.09	0
	Very poor	0	27.27	27.27
	Unfit for drinking	100	36.36	72.72
RI	Practically uncontaminated	0	63.63	27.27
	Moderately contaminated	0	36.36	72.72
	Heavily contaminated	63.63	0	0
	Extremely contaminated	36.36	0	0

### Nutrient status assessment by Trophic State Index (TSI)

**Figure 4:**
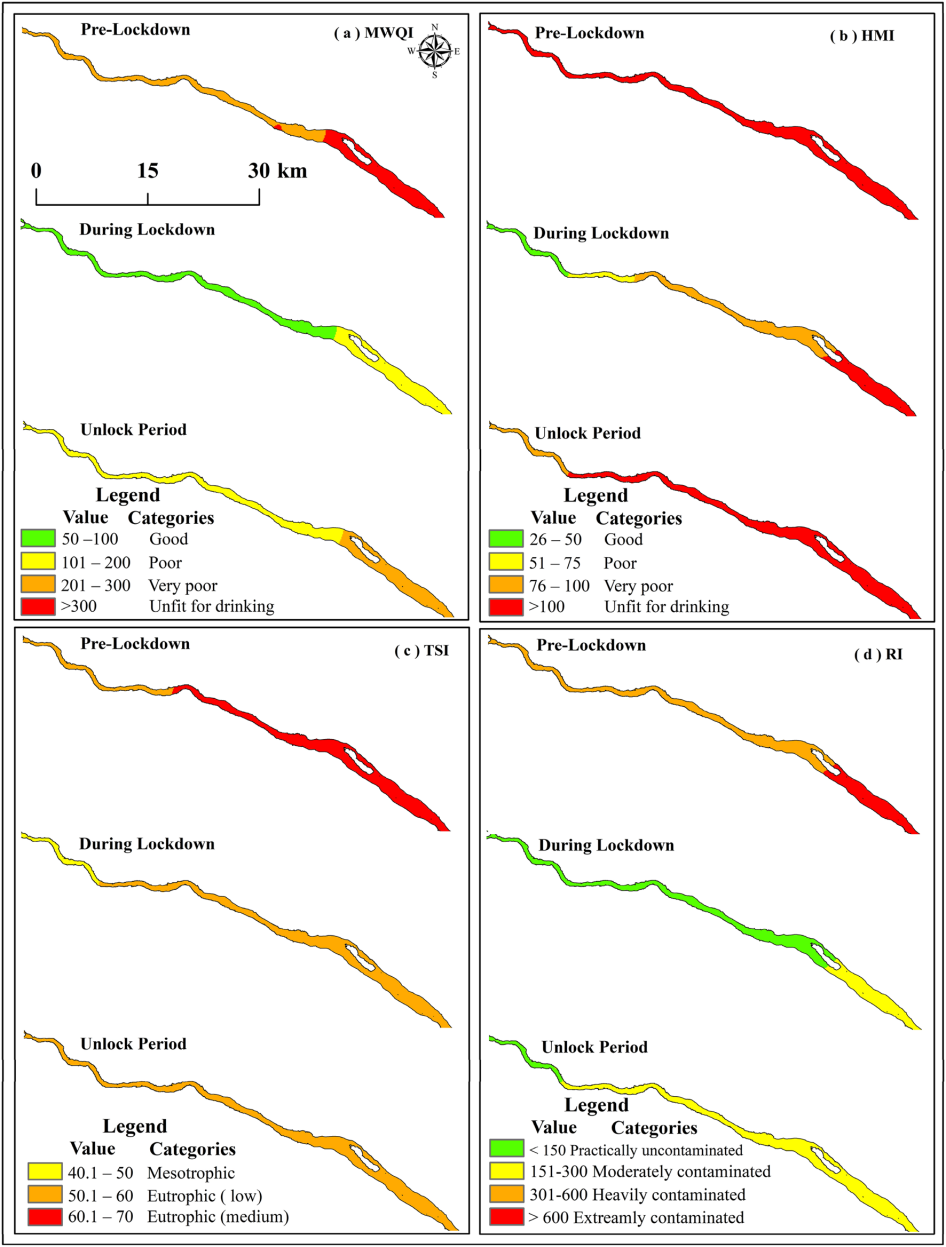
Spatial variation of water quality in three periods based on different water quality index. The diagram is prepared by open. source QGIS 3.16 software (https://qgis.org/en/site/forusers/download.html).

TSI assessment of pre-lockdown phase showed that the values ranged from 57.53 to 65.98 with mean and SD 61.99 ± 2.63. 36.36% samples showed low eutrophic status and 63.63% was the moderate eutrophic status (Tables 3, 4). During lockdown phase, low concentration of Chl-*a* and total phosphorus (TP) helped to lower down the tropic level of water and the value ranged from 44.89 to 55.70 with a mean and SD of 51.89 ± 3.26. 27.27% samples were mesotrophic status and 72.72% samples were low eutrophic status (Table 4). Duringunlock phase 100% samples identified eutrophic (low) category of water (Fig. 3b). Amplification of Chl-*a* and TP concentration with low Sd depth helps to promote tropic level of river water and again deterioration of water quality. The spatiotemporal variations of water quality presented in Figs. 3 and 4. Spatial mapping clearly indicated that upstretch of river was less contaminated compared with down stretch.

### Water quality assessment by Heavy metal Index (HMI)

Heavy metal concentration showed that the values of HMI in pre-lockdown phase ranged from 226.80 to 413.67 with a mean and SD of 335.29 ± 59.77 while 100% samples were unfit for drinking purpose (Tables 3, 4). During lockdown phase, HMI value ranges from 33.96 to 117.33 with a mean and SD of 86.06 ± 31.28. The water quality showed 27.27% good, 9.09% poor-, 27.27% very poor, and 36.36% unfit for drinking purposes (Fig. 3c). HMI was declined by − 74.33 during lockdown phase and it rose to -58.24 during unlock phase compared with pre-lockdown phase (December 2019) (Table 3). Unlock phase, HMI showed that the values ranged from 89.32 to 175.06 and 27.27% samples were very poor quality while 72.72% samples were unfit for drinking purpose. Figure 3c is representing individual sampling sites with the heavy metal concentrations of three phases.

### Potential ecological risk assessment index (RI)

In pre-lockdown phase, potential ecological risk index (RI) values showed that it ranges from 411.03 to 654.30 with a mean and SD of 537.45 ± 87.19, which indicated moderately contamination by its mean. 63.63% water samples showed highly contaminated whereas 36.36% represented moderately contaminated of the aquatic environment (Table 4). During lockdown phase, RI of toxic metals showed the range 60.42–171.67 with a mean and SD of 124.53 ± 44.20. In this phase, around 63.63% of water samples were practically uncontaminated and about 36.36% were moderately uncontaminated river ecosystem (Fig. 3d). After reopening of public activities in unlock phase, RI showed the range from 140.45 to 248.30 with mean and SD values of 194.91 ± 42.17; and around 27.27% of water samples showed practically uncontaminated and 72.72% samples illustrated moderately contamination to potential ecological risk of the aquatic environment. RI value was dropped by − 76.83% during lockdown phase and again − 63.73% declined during unlock phase compared to pre-lockdown phase (December 2019) (Table 3). The Spatiotemporal variations of potential ecological risk presented in Figs. 3 and 4.

## Discussion

In the twenty-first century, excessive population pressure, irrational land use practice and anthropogenic pollution are vital problems in the global scale and the crucial effects are reflected on aquatic environment as well as river health^10,11^. Urban-Industrial pollution has become a principal reason for the deterioration of river water quality (RWQ). Rapid urbanization and industrialization are root causes of river pollution and resulted in a significant rise in sewage generation and untreated wastewater on riverbed across the country. However, before the sudden emergence and outburst of the Covid-19 pandemic, allnatural and anthropogenic activities were continued by normal rhythm. During the pre-lockdown phase, intensification of various human development projects has been generated huge pollution loads and these are dumped into nature^10,11,36^. Due to Covid-19 pandemic situation, India took the decision of sudden full lockdown and it was effected from 25th March, 2020 and it continued on 31st May 2020. Meanwhile, a short period restriction of industrial sectors and economic activities offered an opportunity to restore the river water quality (RWQ) or river health itself from the usual exploitation by urban-industrial activities^11^.

The present study stretch of river Damodar is surrounded by various large and medium scale industries, thermal power plant, dense settlement, large urban areas and agricultural fields etc. During pre lockdown, the untreated industrial effluents (solid/liquid) are directly discharged into river and these are mixed with river water. This study identified 11 continuous discharges of waste effluent sites while industries have not followed any pollution guide lines and acts. A metadata analysis showed that all physico-chemical and biological water quality parameters were much higher than their permissible limit as indicated by WHO^32^ and BIS^31^ during pre lockdown and unlocks phases. Higher concentration of chloride and sulphate indicated regular mixing of solid and liquid waste water from nearby industries during pre-lockdown and unlock phase. It also indicated the very high mixing of inorganic chloride matters to the riverbed by iron and steel industries and sponge iron factories which are situated beside river bank. Pollutants and heavy metals are transported by runoff water from coal mining fields and these were mixed up with river water at sample site S1, which contributed significant pollution in river water^37^. S2 location was highly influenced by waste effluents of nearby sponge iron factory and therefore, high concentration of all parameters has been found in this location. Sample sites like S3, S4 and S5 were getting polluted by cement factories, iron and steel plant and sponge iron factories, respectively. Therefore, high abundance of hot waste water, untreated effluents and contained heavy metals was considerably found at these locations. Supply of magnesium, chloride, sulphate promotes ionic activities in river water and it leads to unsuitable for human consumption. Hot waste water and mixing of heavy metals in river water promotes growth of aquatic microorganisms. Bacteria in water consume dissolved O_2_ and release dissolved CO_2_. It positively helps to create such a favourable condition for algae growth^38^. In all sample sites of river Damodar, richness of Chl-*a* has been found very high in pre-lockdown phase. Sample sites S6, S7 and S8 were influenced by waste effluents of thermal power plant, cement factories along with urban runoff of domestic discharge from nearby Raniganj, Durgapur cities. Sampling sites S9, S10 and S11 were highly contaminated by solid and liquid discharge of steel plant, chemical industries and urban effluents and nearby agricultural runoff. High abundance of all physico-chemical and biological factors has been observed at the sampling sites. Many previous researches on water quality of river Damodar also indicated that untreated sewage and toxic effluents of industries were the main cause of water quality deterioration of river Damodar 11–12^20,22^.

Evident from the recent studies reveal that the river water quality has been revived significantly during lockdown phase. Due to complete closing of industrial, transport, business sectors stopped waste discharge admixing to riverbed directly. Statistical analysis showed much lower mean concentration level of water pollutants during lockdown phase. PCA showed that complete lockdown of industrial activities helped to lower down heavy metal concentration in river water. The analysis of variance (ANOVA) strongly accepted significant changes of variables by their mean concentration in three phases. During lockdown, assessment of water quality by MWQI, TSI, HMI and RI indicated significant improvement in river water quality (RWQ) mainly due to closing of industrial activities and during lockdown, river water is potentially more suitable for drinking or irrigation purpose compared with per-lockdown phase (December 2019) (Fig. 3).

River water quality (RWQ) is very much important for aquatic ecosystem and riparian organisms. Various numbers of zooplankton, phytoplankton, bacteria, fishes, mollusc types living components build their habitat in aquatic environments^10^. Therefore, biologically it is a very sensitive and diversified area that maintains the natural cycle of ecosystem. Riverbed and riparian environments are highly dependent on various physico-chemical and biological parameters. Any changes on one of them can cause serious environmental hazards to the entire biotic community of water. Not only that, human and other animal species, plant species could get hampered by ecological deterioration of water quality. Toxic heavy metals are one of the main causes of environmental hazards to aquatic ecosystem. Heavy metals can transfer among many communities by food chain and directly affects the physiological system of biotic communities. In this study, we assessed the effects of anthropogenic activities on RWQ by ecological sensitivity analysis. The potential ecological risk (RI) of pre-lockdown indicated moderately contaminated (36.36%) to heavily contaminated (63.63%) by heavy metals in river water. During lockdown, very low supply of toxic metals helped to reduce environmental risk as it changes from practically uncontaminated (63.63%) to moderately contaminated (36.36%) condition. After restarting of all industrial, commercial activities increased heavy metals supply again to riverbed and reduce practically uncontaminated (27.27%) and increased moderately contaminated (72.72%) water in River Damodar. Figures 3 and 4 present the spatiotemporal variation and sample site-wise distribution in RWQ corresponding to different study phases.

## Conclusion

In the present study, the river water quality of Damodar of an urban-industrial belt area has been analyzed to comprehend the anthropogenic impact during Covid-19 lockdown (June, 2020) in compared with pre-lockdown (December 2019), and unlock phase (November 2020). A metadata analysis of river water quality (RWQ) variables and various indexing methods indicates that the maximum variables were declined in lockdown phase in compared with pre-lockdown phase. Due to complete stopping of activities of industries, mining, commercial sectors highly helped to improve water quality by no mixing of waste effluents directly discharged to the river water.

**Figure 5:**
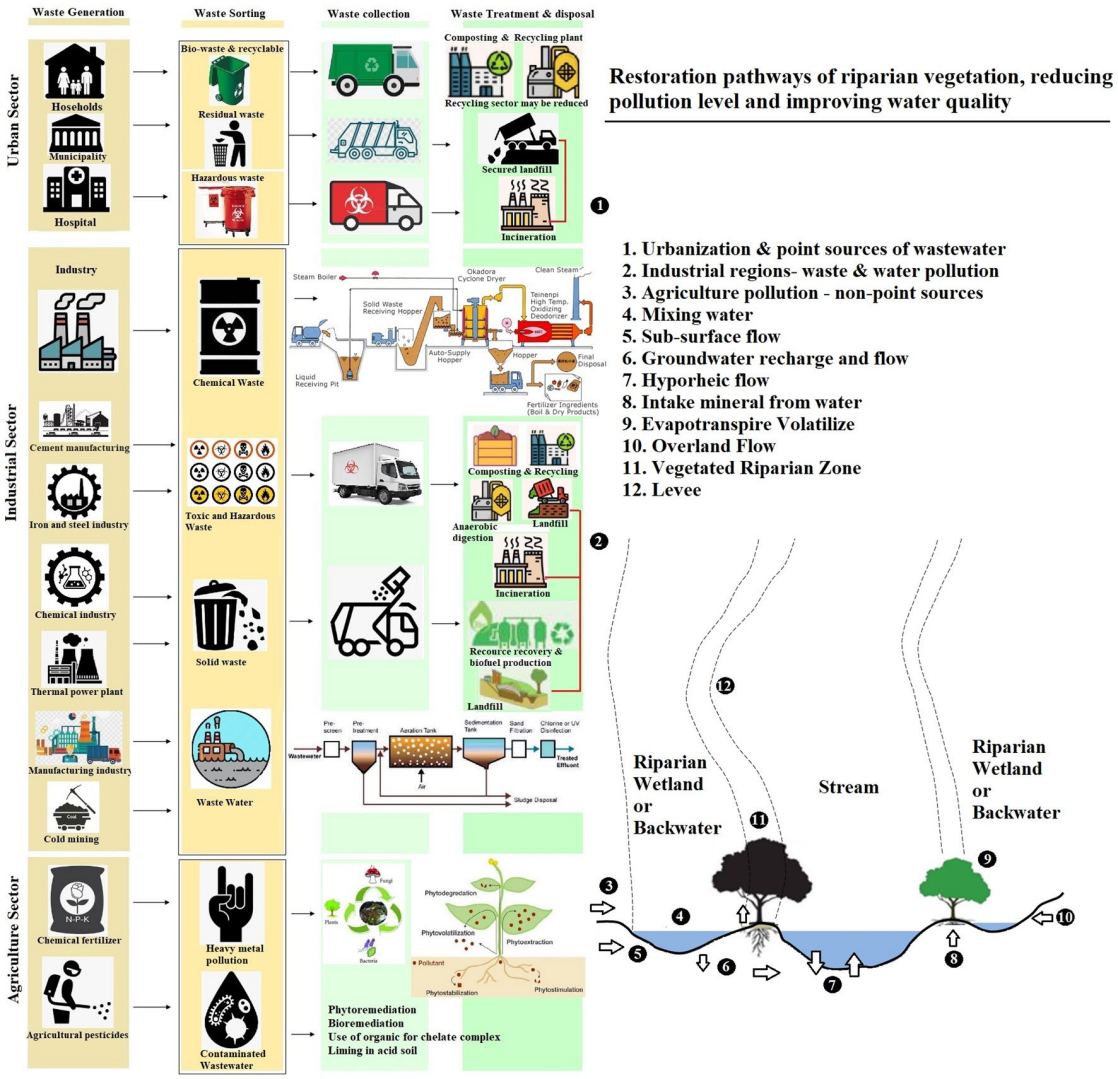
Eco-restoration and management plan for reducing pollution and improving river water quality (RWQ). This figure is prepared by MS office 2007 (https://microsoft-office-2007.en.softonic.com/).

It is indubitably evidenced from this assessment, unlock phase (November, 2020) of every public and private sectors especially industrial and commercial activities again started waste discharge to river water directly and consequently, RWQ is going deteriorated like pre-lockdown phase. In this circumstance, a suitable management framework and scientific remediation should be very helpful to restore river water quality without prohibition of human developmental activities in this new normal era. We have proposed a rational management framework and guide to improve river water quality (RWQ) as well as restore river health (Fig. 5). Regular monitoring of river health will definitely reduce the huge anthropogenic stress and recovers the river resilience, water quality, structure and ecological functions. We recommended that the relevant government regulations and local administrative decisions should be implemented and it is more applicable to control river water pollution and improving river water quality and opportunity for aquatic environments to heal it.

## Supplementary Information

Supplementary Information.
