# Supplementary material for: Positive effects of COVID-19 lockdown on river water quality: evidence from River Damodar, India
Source: Sci Rep. 2021 Oct 11;11:20140. doi: 10.1038/s41598-021-99689-9 (PMC8505400; doi:10.1038/s41598-021-99689-9)
Supplement: Supplementary file 1 — Supplementary Information. [file 41598_2021_99689_MOESM1_ESM.pdf]

Table S1: One way ANOVA analysis

Variable		Sum of Squares	df	Mean Square	F	Significance level
pH	Between Groups	1.996	2	0.998	4.861	0.015
	Within Groups	6.160	30	0.205		
	Total	8.157	32			
TDS (mg/l)	Between Groups	269553.316	2	134776.658	72.868	0.000
	Within Groups	55487.991	30	1849.600		
	Total	325041.307	32			
Turbidity (NTU/l)	Between Groups	1276.788	2	638.394	29.865	0.000
	Within Groups	641.273	30	21.376		
	Total	1918.061	32			
EC (µg/l)	Between Groups	658089.152	2	329044.576	72.868	0.000
	Within Groups	135468.727	30	4515.624		
	Total	793557.879	32			
Mg^{2+}(mg/l)	Between Groups	10367.697	2	5183.848	100.450	0.000
	Within Groups	1548.182	30	51.606		
	Total	11915.879	32			
Ca^{2+}(mg/l)	Between Groups	24174.970	2	12087.485	19.860	0.000
	Within Groups	18259.273	30	608.642		
	Total	42434.242	32			
Cl^{-} (mg/l)	Between Groups	251145.455	2	125572.727	71.006	0.000
	Within Groups	53054.545	30	1768.485		
	Total	304200.000	32			
So_4^{2-}(mg/l)	Between Groups	307218.182	2	153609.091	60.737	0.000
	Within Groups	75872.727	30	2529.091		
	Total	383090.909	32			
No_3^{-}(mg/l)	Between Groups	13238.631	2	6619.315	53.513	0.000
	Within Groups	3710.858	30	123.695		
	Total	16949.489	32			
BOD(mg/l)	Between Groups	152.970	2	76.485	9.086	0.001
	Within Groups	252.545	30	8.418		
	Total	405.515	32			
DO(mg/l)	Between Groups	58.439	2	29.219	3.404	0.047
	Within Groups	257.526	30	8.584		
	Total	315.964	32			
Zn^{2+} (µg/l)	Between Groups	8215036660.606	2	4107518330.303	303.727	0.000
	Within Groups	405711527.273	30	13523717.576		
	Total	8620748187.879	32			
Cd^{2+}(µg/l)	Between Groups	296.209	2	148.105	67.569	0.000
	Within Groups	65.757	30	2.192		
	Total	361.966	32			
Pb^{2+}(µg/l)	Between Groups	3481.692	2	1740.846	160.305	0.000
	Within Groups	325.787	30	10.860		
	Total	3807.479	32			
Ni^{2+}(µg/l)	Between Groups	37402.909	2	18701.455	48.888	0.000
	Within Groups	11476.000	30	382.533		
	Total	48878.909	32			
Cr(µg/l)	Between Groups	18749.385	2	9374.693	133.717	0.000
	Within Groups	2103.254	30	70.108		
	Total	20852.639	32			
Total Fe(µg/l)	Between Groups	1719881.879	2	859940.939	130.291	0.000
	Within Groups	198005.091	30	6600.170		
	Total	1917886.970	32			
SD	Between Groups	1.701	2	0.851	4.181	0.025
	Within Groups	6.103	30	0.203		
	Total	7.804	32			
Chl a(mg/l)	Between Groups	12181.091	2	6090.545	41.161	0.000
	Within Groups	4439.091	30	147.970		
	Total	16620.182	32			
TP	Between Groups	11094.606	2	5547.303	42.877	0.000
	Within Groups	3881.273	30	129.376		
	Total	14975.879	32			

Table S2: Varimax rotation component matrix of factor loadings in pre lockdown, during lockdown and after unlock period

Pre lockdown				During lockdown					After unlock			
Factor loading	1	2	3	Factor loading	1	2	3	4	Factor loading	1	2	3
No3	0.907	0.223	0.087	Pb	0.945	0.156	0.2	0.146	TDS	0.906	0.247	0.055
Po4	0.829	0.446	0.186	Turbidity	0.935	0.097	0.206	0.252	EC	-0.902	-0.069	0.311
Cl	0.826	0.486	-0.003	Cr	0.931	0.316	0.083	0.014	Turbidity	0.842	0.481	0.229
Fe	-0.82	-0.309	-0.303	Po4	0.914	0.244	0.251	0.172	Pb	0.84	0.493	0.043
Cd	0.765	0.511	0.306	Cd	0.907	0.23	0.279	0.162	Ca2	0.815	0.539	0.181
Ca2	0.764	0.536	0.26	Ni	0.866	-0.012	0.159	0.465	Cd	0.758	0.619	0.017
Zn	0.748	0.569	0.291	Chl	0.839	0.386	0.307	0.141	So4	0.735	0.666	0.112
Sd	0.735	0.043	0.386	No3	0.835	0.303	0.374	0.226	Po4	0.706	0.695	0.102
Ni	0.73	0.608	0.221	Zn	0.802	0.143	0.207	0.507	Zn	0.697	0.424	-0.252
Pb	0.712	0.631	0.161	Cl	0.675	0.575	0.378	0.016	BOD	0.687	0.683	0.118
Turbidity	0.706	0.55	0.328	Sd	-0.642	-0.12	-0.514	-0.437	Cr	0.683	0.661	0.087
Mg2	0.339	0.804	0.01	So4	0.628	0.44	0.379	0.352	Ni	0.072	0.934	0.146
DO	0.619	0.766	-0.049	Fe	0.628	0.361	0.382	0.406	pH	0.294	0.886	0.024
pH	0.148	0.747	0.223	TDS	0.204	0.944	0.008	0.046	Mg2	0.545	0.808	0.04
So4	0.448	0.731	0.06	EC	0.204	0.944	0.008	0.046	No3	0.614	0.77	0.087
Cr	0.61	0.7	0.194	Ca2	0.07	0.836	0.379	-0.092	Cl	0.644	0.721	0.03
Chl	0.584	0.668	0.059	pH	0.248	0.693	0.174	0.389	Chl	0.695	0.704	0.112
TDS	0.343	0.042	0.929	DO	-0.301	-0.208	-0.893	-0.147	Fe	0.666	0.667	0.079
EC	0.343	0.042	0.929	BOD	0.552	0.137	0.673	0.111	Sd	-0.534	-0.659	0.176
BOD	-0.201	0.591	0.719	Mg2	0.29	0.044	0.116	0.925	DO	0.002	0.116	0.983
Eigen value	14.014	2.12	1.444	Eigen value	13.723	2.637	1.092	1.008	Eigen value	15.866	1.638	1.02
% of variance	70.069	10.599	7.22	% of variance	68.617	13.185	5.458	5.038	% of variance	79.331	8.192	5.099
Cumulative variance	70.069	80.668	87.888	Cumulative variance	68.617	81.802	87.26	92.298	Cumulative variance	79.331	87.523	92.622
